# Supplementary material for: Are carbon emissions trading and green financial instruments synergistic? -Comprehensive quantitative research based on content analysis
Source: PLoS One. 2024 Mar 7;19(3):e0298601. doi: 10.1371/journal.pone.0298601 (PMC10919601; doi:10.1371/journal.pone.0298601)
Supplement: S1 Data — (ZIP) [file pone.0298601.s001.zip › Data/地级市Z_F工作报告.zip!/╣π╬≈/╖└│╟╕█╩╨/└┤╫╘╣1⁄2╓┌║┼í╛┬φ┐╦╩2╛▌═°í┐_2009.docx]

各位代表：

现在，我代表市人民政府向大会作政府工作报告，请予审议，并请各位政协委员和其他列席会议的同志提出意见。

2008年工作回顾

2008年是大事多、喜事多的一年，是极不平凡、极其重要的一年，是面对严重自然灾害和国际金融危机的一年。我们全面落实科学发展观，认真贯彻党的十七大、十七届三中全会和胡锦涛总书记、吴邦国委员长视察广西及温家宝总理、张德江副总理亲临我市的重要指示精神，切实按照郭声琨书记、马飚主席对我市的战略定位和发展要求，在市委的正确领导下，紧紧抓住《广西北部湾经济区发展规划》批准实施和钢铁、核电项目落户等重大历史机遇，落实市委提出的“两个定位”、“三个兴市”、“四大产业”、“五大基地”和刻苦攻坚跨越发展三年计划的战略部署，积极应对国际国内复杂形势和重大自然灾害的严峻挑战，全面对接国家发展战略，举全市之力推进钢铁、核电两个特大项目的前期工作，大力实施“项目建设推进年”，掀起新一轮大开发、大建设热潮，促进全市经济社会又好又快发展，较好地完成了市四届人大四次会议确定的目标任务，大事办成了，喜事办好了，难事克服了，人气兴旺了，主要经济指标增幅保持在全区前列，各项社会事业全面发展，边疆巩固安宁，社会和谐稳定，人民安居乐业，防城港市的发展掀开了崭新的一页。

经济社会发展保持良好势头，主要指标实现历史性突破。初步统计，全市生产总值、财政收入、规模以上工业总产值、金融机构本外币存贷款余额、房地产投资等指标增幅名列全区第一，外贸进出口总额、港口货物吞吐量、海关税收、资金净流入量、农民人均纯收入等指标总量位居全区第一。全市生产总值突破２００亿元，达到２１２．１８亿元，增长２０．１％，实现连续８个季度２０％以上的快速增长；人均ＧＤＰ突破３５００美元；财政收入突破２０亿元，达到２１．９２亿元，增长３９．１％；全部工业总产值突破３００亿元，达到３２７．５１亿元，增长４３．９％，规模以上工业总产值突破２００亿元，达到２７７．１８亿元，增长５０．９％；年末金融机构存款余额１７３．８２亿元，比年初增长３７．７％，年末贷款余额８３．４２亿元，比年初增长２９．４％；房地产完成投资２９．２２亿元，增长７８％；外贸进出口总额完成２２．０７亿美元，占广西的六分之一强，增长５１．３％；港口货物吞吐量完成５６２６万吨，占广西沿海港口总量三分之二强，增长１１．３％；海关税收６７．９４亿元，占广西的６２．１％，增长４０．３％；已成为广西资金净流入最多的市，全年达到１８５亿元，占全区的２３．５％；农民人均纯收入４４７４元，增长１８％。城镇居民人均可支配收入１４３６４元，增长１８．１％。全社会固定资产投资完成１４６．０５亿元，增长４１．１％。

三次产业协调发展，经济发展支撑力明显增强。产业结构进一步优化，三次产业比重由２００７年的２０∶４７．１∶３２．９调整为１７∶４９．９∶３３．１，工业高速增长，主导地位进一步增强。防城港电厂一期工程投入商业运行，益海大豆浓缩蛋白、农垦防城分公司精制糖等一批工业项目顺利投产；粮油加工、冶金、制糖、化工等重点行业快速发展，其中粮油加工产值超１３０亿元，钢铁产值超３０亿元；产值超亿元的企业达到３６家，同比增加７家。交通运输、金融服务、房地产等第三产业发展加快，有效促进了消费需求，社会消费品零售总额完成３８．１５亿元，增长２３．７％。社会主义新农村建设扎实推进，县域经济实力不断增强。东兴市、防城区被评为“２００７年度广西经济发展十佳县（市、区）”。全市农业总产值５８．６亿元，增长５．１％。县域生产总值１３９．６亿元，增长１８％，占全市生产总值６５．８％。经济林、糖料蔗和服务大项目的种植、养殖基地发展迅速。

全面对接北部湾发展规划，总体发展思路更加明确。结合开展继续解放思想大讨论活动，全面对接《广西北部湾经济区发展规划》，调整和完善了总体发展思路。以“滨海、门户、生态”和“组团式”城市等新理念，确定了“三岛三湾一核心六组团”的城市新格局，《防城港市城市总体规划》已上报自治区人民政府审批。经积极谋划，中心城区规划面积由原定的７０平方公里增至１４０平方公里，人口由５０—６０万增至超１００万，极大拓展了城市发展空间。积极配合自治区做好北部湾经济区城镇群、大型组合港建设等专项规划。重点编制和修编了企沙工业区总体规划、市旅游业发展总体规划、土地利用总体规划、海洋功能区划等３０多项重要规划。

钢铁核电项目取得重大突破，奠定跨越发展基础。我市乃至全区各族人民期盼已久，有史以来投资最大，一期总投资６８６亿元的防城港钢铁基地项目和装机６００万千瓦、总投资近７００亿元的红沙核电项目分别在２００８年３月１７日和１０月１０日获得国家同意开展前期工作。市委、市政府迅速成立钢铁核电项目推进工作总指挥部，举全市之力把两大项目作为“天字号工程”奋力推进。强化组织协调，坚持前期工作、现场工作、后勤保障、社会治安统筹推进。积极配合自治区做好用地用海、环保评价、水土保持、岸线利用等前期工作，项目报批核准工作基本完成。实行市四家班子领导和部门包干负责制，在中直、区直单位积极参与和各区（县、市）大力支持下，在全市范围内抽调上千名干部驻村入户，开展深入细致的群众工作，全面推进征地拆迁安置，仅用５个多月就完成了钢铁项目７３００多亩９００多户的征地拆迁安置任务；企沙工业区“两区域两支撑”项目加快推进，水电路网全面开工，现场数十座山头已夷为平地，上下游配套产业规划编制及招商引资工作顺利启动，重型机械制造及修造船等一批项目在抓紧洽谈落实。核电项目征地拆迁协议签订已超过８０％，仅用２个月就基本贯通了核电进厂道路，１２月２０日自治区隆重举行了核电项目前期工程启动仪式。１６个配套社会事业项目前期工作和地方建材、副食品供应等后勤保障工作扎实推进。在征地拆迁安置过程中，我们统筹城乡发展，关注群众利益，出台利民政策，实现了“文明搬迁、和谐搬迁”。两大项目的快速平稳推进，创造了“防城港速度”，得到了自治区党委、政府和区内外社会各界的充分肯定。

“项目建设推进年”活动扎实开展，发展后劲不断增强。坚持大中小项目并举等“五个并举”和市四家班子分工负责制，强化督促落实和检查协调，实现了项目建设新突破。投资３０００万元以上的重大项目已开工６６项，建成或基本建成２０项。沿海基础设施建设大会战一期项目基本完成，防城南站至企沙铁路等二期实施项目完成投资８８％。市污水处理厂、东兴市给水工程等一批自治区５０周年大庆项目顺利推进。投融资工作取得重大突破。成立了市融资工作组，发挥城投公司、土地储备中心等市本级融资平台的作用，落实了７亿多元贷款，与开行、建行初步达成６００亿元合作意向。与广西北部湾国际港务集团、广西投资集团、上海宝冶建设有限公司等大型国企建立投资合作关系。集中市本级２０００万元财力有效推进一批重大项目前期工作。工程招投标和投资评审等制度进一步规范，核减资金２．０８亿元。

城市建设全面展开，城区面貌明显改观。以城市中心区为重点的城市建设全面拉开大幕，东西大道、南北大道、迎宾路和西湾广场周边街道亮化绿化工程已竣工，兴港大道改造工程、中心区环路工程、阳光海岸、金海湾商住区、仙人山公园、红树林保护工程等一批项目开工建设。市中级法院、检察院进驻新址办公。防城区防港路、防钦路等道路整治改造工作基本完成，大西南商贸城等一批房地产项目建设加快。上思县、东兴市以及企沙镇、江平镇等城镇化稳步推进。城市建成区面积３４．０９平方公里，同比增长６．２％，城镇化率达４４．５％。运用ＢＴ融资方式吸引近３亿元社会资本参与一批城建项目建设。土地清理整治工作全面推进，历史遗留问题逐步解决。

港口生产建设加快，核心竞争力持续增强。企沙半岛岸线利用重新定位，建设１０亿吨大港列入规划。港口建设完成投资１０亿元。１０万吨级西湾航道工程完成建设任务，１６、１７号５万吨级泊位进入收尾阶段，１８－２２号７－１５万吨级泊位水工工程基本完成，开始设备安装，５０１、５０２号５万吨级泊位等一批新开工项目推进顺利。全市港口货物吞吐量完成５６２６万吨，其中防城港货物吞吐量完成３７０１万吨，增长２２．１％。集装箱吞吐量完成２２．５７万标准箱，增长３０．４％。成功开行防城港至昆明物流中心海铁集装箱联运五定班列。 招商引资力度加大，对外开放水平不断提高。积极参与第五届中国—东盟博览会和商务与投资峰会、第三届泛北部湾经济合作论坛、第四届桂台经贸合作交流会，主动组团赴武汉、大连、唐山曹妃甸、上海、南京、深圳、港澳地区及东盟、日本等地考察推介、招商洽谈，充分利用中越边境（东兴—芒街）商贸·旅游博览会等平台，进一步拓展同境外及国内沿海发达地区的交流与合作。与越南下龙市结为友好城市。建立健全招商引资项目大兑现保障机制，建立联席会议部门会审的项目落户机制，引进区外境内资金４３．９４亿元，增长７９．７％，外商直接投资额５０９５万美元。呈现城市知名度不断提高，海内外政要和重要客商纷至沓来，投资领域和投资项目明显增多，人气越来越旺的喜人局面。

和谐防城港建设取得新突破，各项社会事业全面发展。财政对民生的投入力度不断加大，农林水发展资金增长５５．４％、社会保障和就业资金增长３７．９％、医疗卫生资金增长６７．３％。发放各类支农补贴１４５１８万元，受益农户１０．２８万户；减轻农民负担１４７５９万元，农民人均减负２４６元。落实城市义务教育学生免除杂费政策，实现了城乡义务教育全部免除学杂费。整村推进扶贫开发取得新成效，解决了近３０００名农村贫困人口的温饱问题，减少了农村低收入贫困人口４０００多人。城镇新增就业人数２１３００多人，农村劳动力转移就业新增１９８００多人。全力做好年初冰冻灾害、夏秋“黑格比”台风灾害的防灾救灾工作，灾区民房重建基本完成。组织发动全市向四川汶川地震灾区捐款捐物合计１９００多万元，人均数居全区第一。社会保险覆盖范围进一步扩大，参保人数达到３２．３７万人。钢铁、核电项目被征地农民首次纳入城镇企业职工社保和城市低保范围；做到被征地农民转移培训就业、养老保险与征地拆迁工作同步进行，并在住房保障、就业等方面制定优惠政策；安排上亿元推进安置点建设；向钢铁、核电项目被征地农民提供４００多个公益性岗位，组织培训２８１２人，培训后转移就业１７９７人，办理社会养老保险８０４人，办理城市低保３１８０人，解决被征地农民后顾之忧。提高职工住房公积金标准，规范了公务员津贴补贴，改善了干部职工福利待遇。

市政府年初承诺的８件事关民生的实事如期完成。保障城市低保对象２０７１９人，月人均补差１３１．１元；保障农村低保对象２５９１１人，月人均补差４２．５元，均超额完成预定任务。新型农村合作医疗参合率达８９．３５％，超过自治区８５％的要求；有２４６００人次得到农村医疗救助，城市医疗救助试点工作稳步推进。实施农村公路项目４７个，完成投资１．２亿元；新增农村饮水安全人口３．３６万人；建成沼气池８０９２座；解决边境３公里以内１２０个自然村通广播电视难问题。学校食堂、卫生室、运动场等项目竣工１１４个。完成１０５５户困难归侨危房改造。新开工建设廉租住房４１８套，建成１０８套，实施廉租住房保障３０９３户。市金海湾商住区动工建设。

市职业教育中心、整合医院创“三甲”等社会公益项目抓紧推进。东兴市获得“自治区卫生城市”荣誉称号。开展打击“两抢两盗”犯罪等百日专项行动，保持打击走私高压态势，取得明显成效。深入开展“领导干部公开大接访活动”，带动解决一大批信访问题。安全生产事故总数、伤亡人数、经济损失呈下降趋势。特别是北京奥运会、自治区成立５０周年大庆等重大活动举办期间均未发生影响社会和谐稳定的突出问题和事件。人口计生工作稳步推进，人口自然增长率９．９４‰。国土资源和海洋利用管理等工作力度加大，支撑经济发展的能力明显提高。节能减排工作扎实开展，主要污染物排放量控制在目标范围内。科技、文化、旅游、体育、民族、宗教、外事、侨务、统计、人民防空、国有资产监督管理、物价、地震、气象、档案、修志等工作取得新进展。红十字会、妇女儿童、老龄、残疾人等事业取得新成绩。双拥工作深入开展。精神文明和民主法制建设得到加强。

服务型政府建设取得新成效，发展环境明显改善。大力实施“效能提升工程”，规范化服务型政府建设成效明显，全市作风效能建设活动高质量、高标准地顺利通过自治区检查验收。组织开展全民评议机关效能活动，９０％以上市直单位获优秀等次，评议、整改情况通过媒体向社会公布。加强制度建设，健全了人事管理、机构编制、城市规划、土地储备管理等运作机制。加强部门协调配合，规范行政审批行为，优化政务服务中心办事流程，强化行政效能电子监察，首问责任制等“三项制度”得到全面落实，工作效率明显提高，市本级行政审批事项办理时限平均缩减７天，比自治区的规定减少５０．１４％。防东、沙企一级公路等收费站整合已获自治区批准，改善了通行条件，提升了对外形象。落实优惠政策，清理、废除和完善各种规章制度１１１项，实行挂牌服务，开设企业绿色通道，企业对我市投资环境满意度近８０％，对我市经济发展潜力满意度达８８％。维护政府诚信，市本级财政偿还历史债务资金９７００万元。自觉接受人大和政协监督，共办理并答复市四届人大四次会议期间人大代表提出的议案、建议、批评和意见９６件，办复率１００％；办理并答复市政协四届三次会议期间政协委员提案１４９件，办复率１００％。

各位代表！

过去一年，我市经济社会发展取得的成绩来之不易！这是党中央、国务院亲切关怀的结果，是自治区党委、自治区人民政府和市委正确领导的结果，是市人大、市政协有效监督和大力支持的结果，是中直、区直单位和驻我市部队支持帮助的结果，是全市广大干部群众和各族各界人士抢抓机遇谋发展、放开思想想问题、放开手脚干事业、争当开放开发排头兵的结果。在此，我谨代表市人民政府，向全市广大干部群众，向解放军指战员、武警官兵和公安民警，向各民主党派、工商联、无党派人士、人民团体，向所有关心、支持、参与我市现代化建设的各界人士和海内外朋友，表示衷心的感谢和崇高的敬意！

在充分肯定成绩的同时，我们也要清醒地看到，经济社会发展中一些事关发展全局的问题尚未得到根本性解决，主要表现在：一是经济总量不大、实力不强的问题依然突出；二是城市功能不全、基础设施落后，“有港无市”的问题依然突出；三是缺乏主导产业支撑和大型骨干企业带动，抵御风险能力不强的问题依然突出。同时，还存在着建设资金筹措难、一些项目推进不够快，节能减排压力大，高层次高技能人才缺乏和个别部门行政效能不高、部分干部服务意识不强等问题。对这些问题，我们必须高度重视，在今后工作中努力加以解决。

２００９年工作总体要求和奋斗目标

今年是新中国成立６０周年，也是我市实施刻苦攻坚跨越发展三年计划、加快实现“十一五”规划目标的关键一年。由于国际金融形势异常严峻，何时见底难以预料，今年制约经济发展的不确定因素增多，金融危机的负面作用日益凸显，我市面临着许多前所未有的困难。去年四季度以来，港口货源减少，货物滞港严重，直接影响港口吞吐量增长，延缓了向亿吨大港快速迈进的步伐。新投产的工业项目不多，部分工业产能受限，食用油、蔗糖等产品价格下跌，企业利润大幅下滑，工业高速增长带动经济快速发展的格局面临严峻挑战。边境小额贸易税收政策调整和增值税转型，将使今年财政收入同比减收３亿元以上，财政收入高速增长的态势已经改变；同时，为了扩大内需和支持项目建设，财政支出的压力更大。农民工失业返乡和重大项目被征地农民增多，导致社会保障、就业安排和社会稳定压力增大。粤港澳等沿海发达地区企业效益下滑，导致民间资本流动性不足，我市承接产业转移和招商引资难度加大。

在充分估计和清醒认识今年我市面临严峻形势的同时，我们更要看到加快发展的积极因素和有利条件，看到危机中蕴含的许多重大发展机遇，增强战胜困难的信心和决心。一是我国经济平稳较快增长的基本面和长期趋势没有变。国家实行积极的财政政策和适度宽松的货币政策，投资４万亿元刺激经济增长，出台一系列扩大内需、促进增长的政策，效应逐步显现。自治区实行６０００亿元的超常规投资计划，把今后几年要开工的项目集中到今年开工建设，继续加快北部湾经济区重大基础设施和重点产业园区建设，这对经济增长将起到极大的促进作用。二是北部湾经济区优先发展的格局没有变。自治区出台了《关于促进广西北部湾经济区开放开发的若干政策规定》，国务院还将专门出台加快广西发展的政策措施。北部湾经济区正奋力前行、加速发展，逐步成为引领、辐射和带动全区加快发展的龙头。三是我市加速崛起、持续快速健康发展的态势没有变。钢铁、核电两个特大项目全面推进的大局已定，大投入大产出促进工业化、城镇化快速发展的格局已定，刻苦攻坚跨越发展三年计划全面实施的步骤已定，加上我市得天独厚的区位优势、港口优势和近几年发展形成的良好基础条件，广大干部群众心齐气顺，对我市经济发展的热情越来越高，信心越来越足，干劲越来越大。这些都为我市跨越发展提供了强力支撑。可以说，这场危机正好给了我们发挥后发优势，发力赶上发达地区的难得机会。防城港市已经站在新的发展起点上！

根据市委的统一部署，今年政府工作的总体要求是：全面贯彻党的十七大和十七届三中全会精神，以邓小平理论和“三个代表”重要思想为指导，深入学习实践科学发展观，紧紧抓住广西北部湾经济区全面开放开发等重大机遇，全面实施刻苦攻坚跨越发展三年计划，以保持经济持续快速增长为首要目标，以扩大内需为基础，以增加投资为保障，以调整结构为主线，以钢铁、核电两大项目为引领，以工业化、城镇化为主导，以改善民生、构建和谐为根本，以“项目建设攻坚年”为主题，大力实施产业发展和城市建设“双百工程”，解放思想，开拓创新，迎难而上，乘势而为，推动经济社会又好又快发展，为加快把我市建设成为广西北部湾经济区生态友好、开放度高、活力迸发的新兴港口工业城市和重要门户城市，实现“一年一个样，五年大变样，十年进入全国沿海开放城市先进行列”的宏伟目标而努力奋斗。

２００９年我市经济社会发展的主要目标为：地区生产总值增长１７％以上；财政收入达到２５亿元以上，增长１４％以上；全社会固定资产投资增长７０％以上；工业总产值增长１８％以上；社会消费品零售总额增长２０％以上；外贸进出口总额增长１０％以上；引进区外境内资金增长３０％以上；外商直接投资增长２０％以上；港口货物吞吐量增长１６％以上；城镇居民人均可支配收入增长１２％以上；农民人均纯收入增长１０％以上；城镇新增就业人数１５０００人，城镇登记失业率控制在４％左右；人口自然增长率控制在１１‰以内；万元生产总值能耗、主要污染物排放量控制在自治区要求范围内。

上述目标中，人口自然增长率、万元生产总值能耗、主要污染物排放量是约束性指标，必须想方设法完成；其他指标是预期性的、指导性的，在实际工作中我们还要朝更高目标迈进。只要我们同心同德，加倍努力，做到“出手要快、出拳要重、措施要准、工作要实”，化压力为动力、化挑战为机遇、化危机为转机、化潜在优势为现实经济增长力，就一定能够有效应对各种复杂局面，就一定能够抓住并用好诸多历史机遇，就一定能够实现科学发展、跨越发展！

２００９年主要工作任务

今年，我们要紧紧围绕中央、自治区关于扩大内需保增长的重大决策，按照市委提出的“促增长、增投资、扩内需、调结构、抓城建、上产业”的要求，坚持产业发展优先、城市建设优先、港口发展优先、可持续发展优先，把大幅度增加投资、上一大批项目作为工作的重中之重，做到“月月有开工，季季有竣工”，以非常信心、非常谋划、非常力度、非常措施、非常办法、非常政策，重点抓好以下工作：

一、坚定不移抓引领，举全市之力继续推进钢铁核电两大项目

钢铁、核电两个特大项目是我市跨越发展的主要希望和根本保障所在。必须举全市之力奋力推进，实现年内全面开工建设。

全力推进征地拆迁安置工作。坚持“文明搬迁、和谐搬迁”，尽快完成钢铁项目配套路网、配套产业区和核电项目厂区、非居住区等征地拆迁安置。安排好临时安置点群众生活，加快推进永久安置区建设。

全力推进主体工程建设。千方百计加大投资，加快推进钢铁项目陆域形成、配套码头、土建、厂房等建设，全面启动炼铁系统高炉、焦化、烧结等工程，加快设备订购，完成海域吹填，完善厂区道路、排水等设施。核电项目要做好现场“四通一平”，加快项目申报、工程准备、设计采购、施工展开、核岛负挖和主体工程开工等各项工作。

全力推进上下游配套项目。围绕建设钢铁能源基地的目标，加大上游和下游配套项目招商力度，开工建设一批上下游产业项目。与广西北部湾经济区管理委员会办公室联合组织力量，开展钢铁项目产业链深度研究，加紧规划千亿元产业集群。加快建设钢铁上游和下游产业区、交通物流支撑、城建与社会事业支撑的“两区域两支撑”基础设施项目。

全力推进服务保障工作。进一步谋划和准备，确保建材、车辆、机械、油料等生产资料和鱼肉禽蛋菜等生活资料的供应。加快建设医疗、教育、宾馆、餐饮及文化娱乐设施，不断提升服务保障能力。

二、实施“产业发展百项工程”，打造临港千亿元产业

经济发展，项目是载体，产业是支撑。要通过五年左右的努力，累计完成投资１０００亿元以上，形成以钢铁项目超千亿元产业为引领，以能源、粮油加工、商贸物流等超百亿元产业为骨干，以及一批几亿到几十亿元产业为配套支撑的产业集群。为此，要大力实施“产业发展百项工程”，突出抓好一批投资额１０００万元以上、对全市发展带动力强的产业项目，今年要完成投资１１０亿元以上，竣工项目２７个，不断增强优势产业对经济增长的支撑作用。

加快发展临港工业。重点发展以钢铁、能源、修造船、重型机械、粮油加工、海产品加工、建材等为主的临海大工业，向千亿元产业集群目标迈进。着力推进防城港电厂二期、重交沥青、风电等项目。今年，粮油加工业力争实现产值超１５０亿元，钢铁产业实现产值３８亿元以上，电力、磷化工、制糖造纸产业实现产值６０亿元以上，建材、矿石、海产品加工业实现产值１３亿元以上。大力实施短平快工业项目，计划投资１４．８亿元，开工９个项目，续建２２个项目，上思华润水泥等项目要在年内竣工投产。

着力扶持企业发展。加大对大海粮油、防城港电厂、盛隆公司等重点企业的扶持，打造一批强优企业。今年大海粮油产值要力争突破１００亿元。加强现有企业的技术和扩能改造，年内完成技改投资２２亿元以上。建立市领导及部门联系帮扶企业制度，切实为企业排忧解难，共度时艰。积极引导企业加强经营管理，大力培育企业品牌，增强产品的市场竞争力，提高企业抵御风险的能力。

加快工业园区建设。按自治区级Ａ类工业园区标准重点抓好企沙工业区建设，尽快完成规划、健全机构，推进项目招商、完善基础设施，以其为龙头，带动东湾粮油加工园区和公车、江平、茅岭、上思城西等工业园区建设。沿海基础设施建设大会战二期项目上半年要全部完工。企沙工业区污水处理厂、自来水厂等重点产业园区项目要尽快开工建设。加快推进钢铁上下游产业配套区路网工程等６个北部湾重点产业园区基础设施项目。抓好防城港海港５００千伏等９个输变电工程和木头滩补水工程、企沙工业区供水安全工程、石板田至东湾工业区供水等一批项目。

三、实施“城市建设百项工程”，全面提升城市综合竞争力

为迅速改变“有港无市”、城镇化明显滞后的局面，必须打响一场城市建设攻坚战，大力实施“城市建设百项工程”，今年要力争完成投资７０亿元以上，竣工３５个项目，实现城市发展新突破。

突出城市发展重点。以中心区开发为核心，充分利用海岛、海湾美丽的自然风光和优良的生态环境，按照“三岛三湾一核心六组团”进行布局，重点抓好“四沿线、四片区、两改造、两景观”，也就是：加快迎宾路、南北大道、东西大道、兴港大道四条道路沿线开发建设，推进行政中心区、桃花湾片区、城南新片区、商业中心片区四个重点片区建设，加大防城城区和渔氵万岛的老城区改造力度，打造西湾景观中心和桃花湖景观中心。同时，推进临江新区开发建设。防城区、港口区的城市建设要积极向行政中心区方向发展。大力发展房地产业，加快建设一批经济适用房和高中低价位相匹配的商品房，年内力争完成投资３０亿元以上。加大政策支持力度，全面落实促进城市建设的优惠政策，鼓励投资者参与城市建设。

抓好城建重大项目。要把沿线和片区建设作为重中之重来抓。迎宾路沿线要推进其沿大酒店、逢时大厦等项目建设；南北大道沿线要推进德城酒店、供电局办公楼等项目建设；东西大道沿线要推进国防大厦、人防指挥中心等项目建设；兴港大道南段改造要在上半年完成，沿线要推进仙人山公园项目一期、海事局综合办公楼、阳光海岸一期等项目建设。行政中心区要突出抓好金海湾商住区等住宅区的开发建设，加快推进中心区环路工程、红树林保护工程、综合写字楼、文化艺术中心、青少年活动中心、科技图书馆、博物馆等一批项目，部分项目年内竣工投入使用。桃花湾片区要加快宁泰夏威夷广场、恒信花园、钢铁项目配套工程等项目建设。城南新片区要推进福满地花园等项目建设。商务中心片区要围绕打造中心商务区的定位，加快站前路等“三横两纵”路网工程、文苑小区等项目建设。开工建设桃花湖景区，加大对西湾景观中心的整治和建设力度。

加快县城和重点镇建设。上思县要以开发新区为主、改造老城区为辅，不断扩大城区面积，增强城市功能。东兴市要积极实施“东扩北移”，开发建设新区，拉开城市框架，开工建设国门广场和口岸改造项目。企沙、江平等重点镇建设也要取得新进展。

加强城市规划与管理。完善“四沿线、四片区”的规划设计。高标准完成临江新区控制性详细规划和江山半岛、西湾景观中心等专项规划。统筹市、区规划和土地管理，按照统一领导、整体规划、综合管理、分工负责、协调高效的原则，建立条块结合、有机衔接的城市建设管理体制，提高城市管理水平。深入开展“城乡清洁工程”，推进城镇绿化、净化、美化工作。做好新建道路的命名，体现城市特色。

四、积极发展第三产业，着力扩大内需

加大政策扶持力度，加快发展现代物流、旅游休闲、金融服务、商贸服务和信息产业等现代服务业，引导第三产业向中心区集中，吸纳人气，催生商机，搞活经济，促进消费。

加快发展现代物流业。尽快完成全市物流总体规划。重点建设东湾临港物流园、冲仑物流园、里火物流园、东兴边民互市区；积极争取国家批准设立防城港保税物流中心（Ｂ型）；大力支持防港物流等一批企业做大做强，拓展仓储、加工、包装、配送等业务，争取建成中国－东盟区域性国际物流基地。

加快发展特色商贸业。尽快启动中心区商业网点的规划建设。抓好金海市场周边商业街改造，精心打造商业中心街、商业精品街、美食一条街等特色商业街。出台政策扶持三星级以上宾馆、大型商场、专业市场建设，尽快规划建设大型农资物流配送中心暨农产品交易批发市场，改造、建设装饰、建材、五金等一批大型专业批发市场和商务园区，开工建设钢铁批发市场和市中心建材专业市场，启动东兴边贸商品集中批发市场建设。尽快规划建设一批加油站。

加快发展特色旅游业。抓紧完成旅游产业发展总体规划，塑造“滨海休闲、港湾运动、边境体验、森林健身、京瑶风情”的旅游新格局。坚持政府主导、市场运作，理顺管理体制，不断加大旅游产业投入。以江山半岛、京岛和西湾景区为重点，抓好大平坡、金滩、十万大山国家森林公园、北仑河口等景区景点建设。推出九龙潭、桃花溪、大板瑶、峒中温泉边境一线特色旅游线路。积极打造京族哈节、国际龙舟节、森林旅游节、金花茶节等旅游文化节庆品牌，提升旅游文化品位。继续办好海滩汽车赛。力争今年启动中越边境旅游异地办证。规划建设旅游信息中心、边境旅游管理中心，改善接待设施，规范景点收费和导游服务，加大宣传促销，不断拓展旅游市场，力争实现旅游人数、旅游收入高速增长，逐步建成国际滨海休闲度假旅游目的地。

加快发展新兴服务业。积极发展钢铁、港口配套产业，大力扶持金融、保险、中介和技术服务等行业，培育我市的设计队伍和项目管理人才，引进一批设计、咨询、工程监理、造价管理、审计、会计等与项目建设密切相关的机构，促进服务业结构优化。

积极引导和促进居民消费。疏通城乡商品流通渠道，抓好家电等工业品下乡活动，加快完善农村商业网点、连锁店。抓紧落实增加城乡居民特别是中低收入群体收入的各项政策措施，增强消费信心和消费能力。鼓励和引导居民扩大住房、汽车、教育、保健等消费，推动消费结构升级，促进消费市场繁荣。

五、扎实抓好“三农”工作，不断壮大县域经济

要认真贯彻落实党的十七届三中全会精神，围绕稳粮、增收、强基础、重民生，强化惠农政策，增强科技支撑，加大投入力度，优化产业结构，推进改革创新，加快社会主义新农村建设，争取做出特色、创出亮点。

深化农村各项改革。深入推进土地流转等各项农村综合改革，努力在重点领域和关键环节改革上实现新突破。全面推进集体林权制度改革，率先在全区完成改革任务。

加快发展特色农业。在稳定粮食生产的同时，大力培育一批特色农产品品牌，做大做强特色优势农业。加大对金花茶、水产品、甘蔗、香料等优势农产品加工业的扶持。以龙头企业带动大宗农林产品基地建设，推动农业规模经营。围绕我市城市发展和钢铁、核电等重大项目建设，调整优化农业结构，着力抓好无公害蔬菜、生态健康养殖、优势水果、特色花卉、绿化苗木等基地建设，大力发展优质高效生态农业和休闲旅游观光农业。加强动物防疫体系建设和食品、药品安全工作。抓好植树造林。加大农民培训力度，千方百计拓宽农民转移就业门路，确保农民收入持续增长。

加强农村基础设施建设。投入２．８亿元加快实施水利建设大会战，重点推进长歧水利灌区、那板水库灌区等节水改造和配套工程建设；加快沙潭江、金海湾等海堤防洪堤建设；抓紧完成１５座中型和重点小一型病险水库除险加固任务；修复一批中小型农田灌溉工程。加大投入，促进农村公路建设。加快企沙渔港建设。增加农村社会事业投入，推动农村公益事业发展。

大力发展县域经济。坚持以特色产业为主导，积极推进县域工业化、城镇化，全面增强县域经济的活力和竞争力。港口区要以港口资源型加工业和现代物流业为发展方向，大力发展重大项目配套产业，把渔洲城工业园区建成全国最主要的磷酸加工出口基地。防城区要大力发展城市经济和城郊经济，努力形成服务大项目的后勤保障基地和全市的教育中心、消费中心、购物中心、娱乐中心。东兴市要充分发挥口岸功能，大力发展外向型经济，加快贸易加工升级和城乡统筹发展步伐，促进旅游、商贸业快速健康发展。上思县要在做大做强糖业、建材、林产、制药等资源型工业的同时，着力打造全市乃至整个北部湾地区的“后花园”。

六、以港口建设为重点，进一步强化出海出边通道建设

紧紧围绕建成亿吨大港的宏伟目标，按照“大型化、专业化、现代化、国际化”的要求，进一步把港口做大做强，率先崛起北部湾经济区的港口龙头，构筑国际大通道。

加快港口发展。全力支持防城港务集团推进１８－２２号泊位、５０１、５０２号泊位、２０万吨级航道、４０３－４０７号泊位和东湾１０万吨级航道等项目。加快亿吨大港建设步伐，充分发挥港务集团对临港产业的集聚带动作用，加大临港工业招商引资项目落户力度，提高本港货源比重；加快东湾物流项目建设，吸引外来物流企业；继续扩大宣传营销，发挥代理公司的作用，开拓国外和西南、中南及区内货源。利用好深水航道和２０万吨泊位，提升对大企业的服务能力。加快白龙、企沙、茅岭、潭油、潭吉等中小港口的对外开放和整合发展，逐步形成大中小港口齐头并进的格局。

加强通道建设。开工建设沿海铁路扩能钦州至防城港段，力争开工建设防城至东兴高速公路、中越北仑河二桥等项目，抓好崇左（经上思）至钦州高速公路、防城至贵台二级公路等项目建设。加快防城至上思一级公路前期工作。推进防城港口岸联检大楼和电子口岸等基础设施建设，提高通关效率和服务质量。尽快完成东兴互市新区、里火互市点、峒中口岸等项目建设。

七、加强改革创新，不断扩大开放合作

要进一步放开思想想问题，放开手脚干事业，坚决贯彻市委提出的“八个更”、“十个只要”等新理念新要求，以超常规思维、超常规办法、超常规力度，冲破一切妨碍发展的旧观念、旧做法、旧体制，开拓创新，破解难题，促进发展。

加大区域合作力度。发挥区位优势，主动融入泛北部湾区域经济，加强北部湾经济区内部的协调合作，扩大与越南下龙、芒街等城市的合作交流，推进东兴边境经济合作区扩大园区提升功能的深度开放，继续办好中越边境（东兴－芒街）商贸·旅游博览会，加快建设国际性重要门户城市。

完善招商引资机制。健全招商项目落户机制，落实项目联席会审制、手续代办制、跟进督查制，实行“保姆式”服务，确保项目快落户、快开工、快建设、快投产。

创新项目工作机制。落实“九大工程”推进责任分工制度，建立健全项目前期工作、融资工作和投资促进工作的联动机制，破解审批、资金、规划、土地等难题，加快推进重大项目实施。抓好重大规划修编。市财政安排５０００万元，组织开展项目前期工作。启动“十二五”重大课题研究和重大项目论证，建立动态备选项目库。清理盘活存量土地，妥善处理历史遗留问题，确保建设用地供给，做好土地收益文章。积极向上争取更多用地指标。

创新投融资机制。做强做大城投公司、土地储备中心、东湾交通投资公司等融资平台，抓好资产注入和抵押等工作，建立健全城市基础设施投融资体制和机制。积极引入战略投资者实施ＢＴ等方式建设，解决场馆建设资金难问题。构建中小企业融资平台，加快中小企业投融资担保体系建设。

做大搞活边境贸易。突出抓好边贸互市，用好国家促进边境贸易发展的财税政策，协调海关等联检部门，积极引导边民参与互市贸易。扶持和鼓励发展一般贸易、加工贸易、服务贸易等贸易方式，支持出口企业深度开拓市场。

推动全民创新创业。积极打造创业平台，营造全民创新创业氛围，以创业促就业、促发展。大力发展非公有制经济。深化人事制度改革，进一步完善人才政策体系，继续推进事业单位人事制度改革。积极帮助城镇新增劳动力、大中专毕业生、下岗失业人员、零就业家庭、被征地农民和返乡农民工就业和创业。

八、抓好财税金融，强化保障支撑

狠抓财源培育。加大税收征收力度，抓好重大项目、重点企业、重点行业的税源培育，做大做强支柱财源，积极开拓边贸互市等新增税源。强化财政收入增长激励机制。充分调动各部门向上争取资金积极性，确保外生财源稳定增长。

加强支出管理。从严控制一般性支出。加大对“双百工程”、重大项目、“三农”工作、市政公共设施、社会保障、节能减排等重点领域和教育、科技、文化、卫生等公共事业的投入。探索建立预算绩效考评体系及机制。进一步健全政府投资评审机制。出台商业银行贷款奖励、扶持工业发展、中小企业融资担保等促进经济发展的激励政策。

加大金融支持。抓住国家实行适度宽松的货币政策的机遇，全力争取和鼓励各银行加大对我市的信贷支持，扩大有效信贷投放，力争新增金融机构贷款１２０亿元。引导各金融机构加强项目对接，促进银政、银企合作。

九、加强社会建设，促进和谐稳定

大力发展社会事业。切实抓好为钢铁基地配套的学校、医院、文化体育场所等１６项社会事业项目。加快推进市职业教育中心建设，积极筹建北部湾职业技术学院和特殊教育学校。实施第四轮创新计划，搭建“产学研”结合的创新平台，提高科技对经济增长的贡献率。实施人才强市战略，在培养和用好现有人才的基础上，大力吸引我市急需的冶金、港口、城建、规划、金融、招商和教育、医疗等方面的专业人才和管理人才。积极打造边山海特色文化品牌，推出一批文化精品。依法推进人口与计生工作，加大治理出生人口性别比偏高的力度，抓好乡镇计生服务所等设施建设。逐步完善公共卫生体系，加强农村三级卫生服务网络建设，做好妇幼卫生保健工作。深入开展全民健身运动，推进市体育综合训练馆建设；抓紧与自治区体育局筹划苏桂南方体育训练基地项目。

强化社会保障。加快完善社会保障体系，继续扩大社会保险覆盖面和受益面，提高保障标准。尤其要完善重大项目征地拆迁安置配套政策。进一步扩大新型农村合作医疗覆盖面，实施城镇居民基本医疗保险制度。抓好市老年活动中心、儿童福利院和残疾人康复服务中心等社保基础设施项目。

确保稳定安全。加强和改进信访工作，继续深入开展“公开大接访”和“领导干部下访”活动。加强社会治安综合治理和边海防管理，坚决打击走私、贩毒、赌博和“两抢两盗”等各种违法犯罪活动。落实国家“安全生产年”工作部署，提高应急管理能力，防止和遏制各类重特大事故发生。

加强节能减排。全面落实节能减排目标责任制，安排专项资金，抓好城镇污水和生活垃圾处理厂等环保设施建设，大力推进节能更新改造，加快淘汰落后产能。加强防城江流域环境综合治理，保护好十万大山和东、西湾生态环境。

抓好双拥等工作。加强国防动员和民兵预备役建设，推进军民双拥共建。进一步巩固和扩大基层民主。充分发挥工会、共青团、妇联等群团组织的桥梁纽带作用。促进民族、宗教、外事、侨务、统计、审计、档案、地方志、新闻出版、防震减灾、人民防空、气象、红十字会、残疾人、妇女儿童、老龄等工作不断发展。

十、坚持以人为本，注重改善民生

要抓住国家扩大内需的时机，将更多财力用于提供与人民生活密切相关的公共产品和公共服务。重点办好９件实事：

１．大力推进被征地农民住宅区建设。加快北港、大龙永久住宅区建设，确保年内竣工；加快建设核电项目被征地农民永久住宅区。

２．加强就业服务。实施农村劳动力职业技能和残疾人就业培训工程；完善征地安置就业机制，提供一批公益性岗位，建立被征地农民就业平台，鼓励和支持被征地农民、返乡农民工自主创业，新增农村劳动力转移就业１．５万人；加强就业中介服务，帮助８００名就业困难人员和１０００名城镇下岗失业人员实现再就业。

３． 加大教育事业投入。实施市高级中学扩建工程；加快边境地区农村义务教育阶段寄宿制学校附属设施建设；改善中小学卫生设施，按规定配备专职医务人员。

４．加强基层文化建设。建成社区文化中心１０个、乡镇综合文化站９个、村文化活动室２４个和“农家书屋”９８个；实施２０户以上通电自然村通广播电视工程，年内完成８８８个村点建设。

５．改善卫生医疗服务。突出抓好市第一人民医院、中医院创“三甲”医院建设；由市、区（县、市）两级财政分担，实行免费婚检和地中海贫血筛查，减少胎儿出生缺陷，促进优生优育。

６．解决困难群体住房问题。投资１．２亿元，建设一批经济适用房和廉租住房工程；完成８００户残疾人危房改造。

７．提高社会保障水平。城市居民最低生活保障月人均补助水平提高到１５０元；农村最低生活保障月人均补助水平提高到４５元；投入７００万元，将边境地区０－３公里范围内１５．５２万农村人口全部纳入最低生活保障范围；进一步落实农村五保户供养政策，提高月人均补助标准；提高城镇“三无”人员集中供养水平；设立爱心救助基金，确保孤寡老人、孤儿、残疾人、贫困学生、失学儿童等困难群体的基本生活。

８．深入实施“兴村富民”工程。抓好２６个村的整村推进扶贫，解决２８００名农村绝对贫困人口温饱问题，减少低收入贫困人口４２００人；投资１．５亿元推进“兴边富民”基础设施建设，改善３－２０公里边境地区农村交通、饮水、茅草房、危房改造、卫生、教育、供电等基础设施；投资２亿多元，实施农村公路项目４５个，新建改建农村公路３８２公里；新建沼气池５０００座；投资３５００万元，推进农村安全饮水工程建设，新增解决农村人口安全饮水３万人以上。

９．实施防城江生态保持工程。按国家补助标准积极筹措资金，建设生态公益林３０万亩以上，大力推进防城江流域水土保持工程。

十一、提高行政效能，构建人民满意政府

新形势、新任务对政府工作提出了新的更高的要求，必须加强政府自身改革和建设，深入实施“行政高效能、项目建设无腐败”行动，不断提升行政能力，着力构建人民满意政府。一要认真开展深入学习实践科学发展观活动，立足于解决实际问题，抓好学习实践活动各阶段工作。加强公务员队伍建设，强化干部教育、培训和管理，不断提高公务员队伍整体素质，适应大开发、大建设的形势需要。开展各级政府、各部门的绩效考评试点工作。二要加强各级政务服务中心建设，扩大政务公开，推进电子政务，强化部门协调配合，提高办事效率，降低创业门槛，减轻企业负担，方便基层办事。三要大兴求真务实之风，深入基层调查研究，强化督促检查，及时解决改革发展中遇到的新矛盾、新问题。坚决反对形式主义，反对急功近利，反对奢侈浪费。四要严格执行《防城港市政府投资工程建设清正廉洁“三十条”》和《防城港市机关作风效能“十不准”》，加强审计、监察，不断完善工程招投标、项目投资评审等管理制度，严把工程质量关、安全关、廉政关，防止“工程建成，干部倒下”的现象发生，以反腐倡廉的成果取信于民。五要坚决执行市委的决策和市人大的决议、决定，自觉接受人大及其常委会的法律监督和政协的民主监督，接受司法监督、社会监督和舆论监督，认真听取各民主党派、工商联、无党派人士和人民团体的意见，完善社会公示、重大事项听证、专家决策咨询和市民意见建议征集等制度，保证群众充分行使知情权、参与权和监督权，提高政府的科学决策、民主决策和依法行政水平。

各位代表！

今年经济社会发展的形势十分严峻，任务十分繁重，责任十分重大。挑战与机遇并存，困难与希望同在。让我们在自治区党委、自治区人民政府和市委的坚强领导下，紧紧咬住刻苦攻坚跨越发展三年计划的目标不放松，团结和依靠全市各族人民，坚定信心、振奋精神，抢抓机遇、应对挑战，只争朝夕、奋力拼搏，全面完成各项任务，以优异的成绩向新中国成立６０周年献礼！
